# LPS-induced release of IL-6 from glia modulates production of IL-1β in a JAK2-dependent manner

**DOI:** 10.1186/1742-2094-9-126

**Published:** 2012-06-14

**Authors:** Aedín M Minogue, James P Barrett, Marina A Lynch

**Affiliations:** 1 Trinity College Institute for Neuroscience, Lloyd building, University of Dublin, Trinity College, College Green, Dublin 2, Ireland

**Keywords:** Glia, Neuroinflammation, SOCS3, STAT1, TNFR1

## Abstract

**Background:**

Compelling evidence has implicated neuroinflammation in the pathogenesis of a number of neurodegenerative conditions. Chronic activation of both astrocytes and microglia leads to excessive secretion of proinflammatory molecules such as TNFα, IL-6 and IL-1β with potentially deleterious consequences for neuronal viability. Many signaling pathways involving the mitogen-activated protein kinases (MAPKs), nuclear factor κB (NFκB) complex and the Janus kinases (JAKs)/signal transducers and activators of transcription (STAT)-1 have been implicated in the secretion of proinflammatory cytokines from glia. We sought to identify signaling kinases responsible for cytokine production and to delineate the complex interactions which govern time-related responses to lipopolysaccharide (LPS).

**Methods:**

We examined the time-related changes in certain signaling events and the release of proinflammatory cytokines from LPS-stimulated co-cultures of astrocytes and microglia isolated from neonatal rats.

**Results:**

TNFα was detected in the supernatant approximately 1 to 2 hours after LPS treatment while IL-1β and IL-6 were detected after 2 to 3 and 4 to 6 hours, respectively. Interestingly, activation of NFκB signaling preceded release of all cytokines while phosphorylation of STAT1 was evident only after 2 hours, indicating that activation of JAK/STAT may be important in the up-regulation of IL-6 production. Additionally, incubation of glia with TNFα induced both phosphorylation of JAK2 and STAT1 and the interaction of JAK2 with the TNFα receptor (TNFR1). Co-treatment of glia with LPS and recombinant IL-6 protein attenuated the LPS-induced release of both TNFα and IL-1β while potentiating the effect of LPS on suppressor of cytokine signaling (SOCS)3 expression and IL-10 release.

**Conclusions:**

These data indicate that TNFα may regulate IL-6 production through activation of JAK/STAT signaling and that the subsequent production of IL-6 may impact on the release of TNFα, IL-1β and IL-10.

## Background

Compelling evidence has implicated neuroinflammation in the pathogenesis of a number of neurodegenerative conditions. Inflammatory processes in the central nervous system (CNS) are mediated by activated glial cells that are capable of producing immunomodulatory molecules, phagocytosing cellular debris and recruiting immune cells from the periphery. Although activation of glia is essential for the maintenance of neuronal function following stress or insult, an uncontrolled response is highly undesirable given the lack of regenerative capacity of the brain.

Microglia are the resident macrophage-like cells of the brain and, as such, play an important role in the maintenance of homeostasis and host cell defense and repair. Astrocytes provide structural, metabolic and trophic support for neurons [1] but they, like microglia, are immunocompetent cells capable of secreting inflammatory mediators. Chronic activation of both cell types leads to excessive secretion of proinflammatory molecules such as TNFα, IL-6 and IL-1β, an effect that may have deleterious consequences for neuronal viability. Indeed enhanced microglial activation, astrogliosis and up-regulation of TNFα, IL-1β and IL-6 expression have been reported in Alzheimer’s disease as well as Parkinson’s disease [2-5].

Microglia and macrophages release TNFα, IL-1β and IL-6 upon activation with the bacterial endotoxin lipopolysaccharide (LPS) *in vitro*[6,7]. The release of these cytokines is mediated by protein tyrosine kinases, mitogen-activated protein kinases (MAPKs) and transcription factors such as nuclear factor кB (NFкB) [8]. However, a specific involvement for each of these kinases in the release of one or other of the cytokines has been difficult to identify.

Type I and Type II cytokines recruit non-receptor tyrosine kinases such as Janus kinases (JAKs) to initiate signal transduction since they lack kinase domains. Interaction of JAK proteins with receptors induces autophosphorylation and causes JAKs to associate with, and activate members of, the signal-transducers and activators of transcription (STAT) which translocate to the nucleus and up-regulate transcription of a number of inflammatory genes [9]. In the present study, we examined the activation of several signaling pathways and the release of proinflammatory cytokines from co-cultures of astrocytes and microglia isolated from neonatal rats in response to LPS. Using flow cytometry, we established that co-cultures contained approximately 80% astrocytes and 16% microglia. Here we show the involvement of JAK2/STAT1 signaling in mediating LPS-induced activation of glia using SAR317461 (formerly TG101209; Sanofi-Aventis, Cambridge, MA USA), a novel specific inhibitor of JAK2.

## Methods

### Materials

LPS (Alexis Biochemicals, Exeter, UK), TNFα (R&D Systems, Abingdon, UK), IL-6 and non-target (NT) siRNA (Invitrogen, Paisley, UK), recombinant IL-6 (R&D Systems), anti-IL-6 receptor and isotype (IgG2b) control antibodies (Biolegend, San Diego, CA, USA), IL-1β Duoset ELISA kit (R&D Systems), IL-6 and TNFα ELISA kits (BD Biosciences, Oxford, UK), anti-phospho-JAK2, JAK2, anti-phospho-JAK1, anti-phospho-STAT1, STAT1, anti-phospho-IκBα and anti-SOCS3 (Cell Signalling, Danvers, MA, USA), anti-phospho-c-jun and anti-TNFα receptor (TNFR1) (Santa Cruz Biotechnology, Heidelberg, Germany, and anti-actin (Sigma, Dorset, UK) were all purchased commercially. SAR317461, a specific JAK2 inhibitor, was a gift from Sanofi-Aventis.

### Primary mixed glial cultures

As astrocytes alone lack sufficient IL-6Rα subunits, and require shedding of IL-6R to mediate IL-6 signaling [10], mixed glial cultures were prepared from the cortices of 1 day old Wistar rats (Trinity College, Dublin, Ireland). Cortical tissue was cross-chopped, incubated for 25 minutes at 37 °C in DMEM (Invitrogen) supplemented with 10% Foetal Bovine Serum (Invitrogen) and 50 U/ml penicillin/streptomycin (Invitrogen) and plated (1 × 10^4^/cm^2^) as previously described [11]. Using flow cytometry, we established that co-cultures contained approximately 80% astrocytes and 16% microglia. After 12 days in culture, cells were pre-treated with a JAK2 inhibitor, SAR317461 (2 μM) for 20 minutes, after which cells were treated with LPS (100 ng/ml) in the presence or absence of SAR317461 for 0 to 24 h. Supernatants were collected and the cells were harvested for analysis of mRNA or lysed for analysis of protein expression.

For co-immunoprecipitation experiments, cells were seeded in 25 cm^2^ flasks and, after 12 days in culture, were treated with TNFα (5 ng/ml) for 0 to 40 minutes. Cell lysates were harvested and the interaction between TNFR1 and the phosphorylated form of JAK2 was assessed by co-immunoprecipitation. To examine the effect of JAK2 inhibition on TNFα-induced changes, cells were treated with TNFα in the presence or absence of SAR317461. Cells and supernatants were harvested at 10 minutes and 6 h, respectively. MTS viability assay (Promega, Southampton, UK) was performed on cells that had been treated with TNFα (5 ng/ml) for up to 24 h.

In another set of experiments, the effect of IL-6 on LPS treatment was assessed in a number of ways: cells were treated with recombinant IL-6 or a neutralizing antibody to the IL-6 receptor or the relevant isotype control (IgG2b); or IL-6 release was inhibited through knock down of the *IL6* gene. Cells were co-incubated for 24 h in the presence or absence of LPS and recombinant IL-6 (20 ng/ml), anti-IL-6 receptor antibody or the isotype control (IgG2b; 100 ng/ml), or either *IL6* siRNA or NT siRNA (50 nM). Supernatants and cells were harvested and assessed for cytokine concentration and mRNA expression, respectively.

### Analysis of IL-1β, IL-6, TNFα and IL-10 concentrations

Supernatant concentrations of IL-1β (R&D Systems), IL-6 and TNFα (BD Biosciences) obtained from glial cultures were measured using ELISA. Cytokine concentrations in the test samples were evaluated with reference to the standard curves prepared using recombinant cytokines of a known concentration.

### Analysis of proteins by western immunoblotting

Western blotting was performed as previously described [12]. Cultured cells were harvested, homogenized in buffer containing Tris–HCl (0.01 M) and ethylenediaminetetraacetic acid (EDTA) (1 mM), and protein (20 μg) was boiled in gel-loading buffer and separated by 7 or 12% sodium dodecyl sulphate-polyacrylamide gel electrophoresis. For co-immunoprecipitation experiments, lysates were harvested and immunoprecipitated using an antibody raised against the TNFR1 prior to separation of proteins on 7% sodium dodecyl sulphate-polyacrylamide gels. Proteins were transferred to nitrocellulose membranes and incubated with antibodies diluted in 5% non-fat dried milk in tris-buffered saline containing 0.05% Tween-20 (TBS-T) against the following: β-actin (1:5000), phospho-JAK2, phospho-STAT1, JAK2, STAT1, phospho-c-jun, anti-SOCS3 and phospho-IκBα (1:1000) for 16 h at 4 °C.

Membranes were incubated with horseradish peroxidise-conjugated secondary antibodies (1:10,000 in 5% non-fat dried milk in TBS-T; Jackson ImmunoResearch, Suffolk, UK) and bands were visualised using Supersignal West Pico Chemiluminescent Substrate (Pierce, Rockford, IL,USA). Images were captured using a Fujifilm LAS-3000 (Brennan and Co, Dublin, Ireland).

### Statistical analysis

Data were analysed using analysis of variance (ANOVA) followed by Newmann Keul’s test or Student’s *t*-test for independent means where appropriate to determine which conditions were significantly different from each other. All experiments were performed three times in triplicate. Data are expressed as means ± SEM.

## Results

### LPS induces activation of JAK/STAT, MAPK and NFκB signaling pathways and proinflammatory cytokine secretion

**Figure 1:**
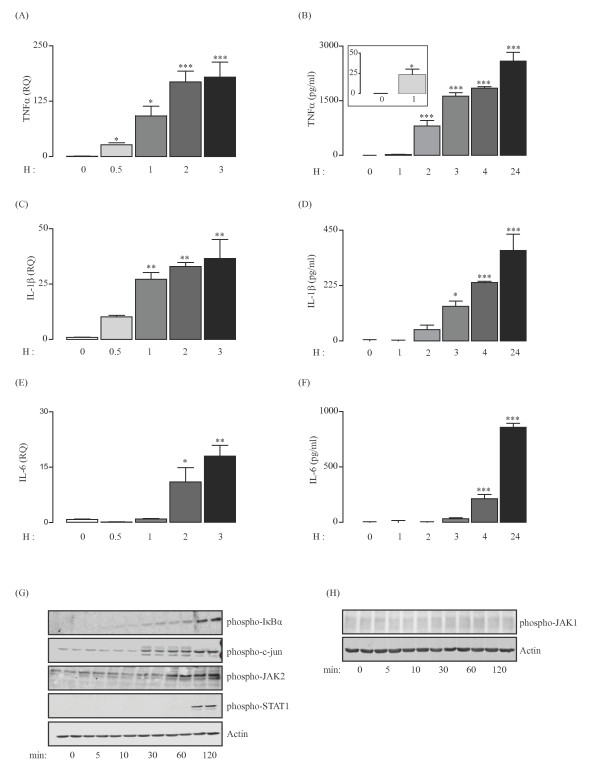
**LPS stimulates activation of JAK/STAT, c-jun and NFкB signaling pathways and release of proinflammatory cytokines from glial cells.** Stimulation of glial cells with LPS (100 ng/ml) enhanced the expression of TNFα mRNA at 30 minutes (**A**; ^*^P < 0.05;ANOVA; n = 3), release of TNFα at 1 h (**B**, see inset; ^*^P < 0.05; Student *t*-test for independent means; n = 3), IL-1β mRNA expression at 1 h (**C**; ^**^P < 0.01; ANOVA; n = 3), IL-1β release at 3 h (**D**; ^*^P < 0.05; ANOVA; n = 3), IL-6 mRNA expression at 2 h (**E**; ^*^P < 0.05; ANOVA; n = 3) and IL-6 release at 4 h (**F**; ^***^P < 0.001; ANOVA; n = 3). Sample immunoblots (representative of three separate experiments) indicate that expression of phosphorylated-IκB, -c-jun, -JAK2 and STAT1 were all enhanced in glial cells incubated with LPS for 10, 30, 60 and 120 minutes (**G**). Expression of phosphorylated JAK1 was unchanged in cells that were treated with LPS (**H**).

The expression and release of cytokines were examined in a time-dependent manner. Incubation of primary glia with LPS (100 ng/ml) stimulated significant increases in TNFα mRNA expression at 30 minutes (*P* < 0.05; ANOVA; Figure 1A) and release of TNFα at 1 h (*P* < 0.05; see inset; Student’s *t*-test for independent means; Figure 1B). IL-1β mRNA expression was increased at 1 h (*P* < 0.01; Student’s *t*-test for independent means; Figure 1C) and IL-1β release was increased at 3 h (*P* < 0.05; ANOVA; Figure 1D). Changes in IL-6 mRNA and release occurred later; IL-6 mRNA expression was significantly increased at 2 h (*P* < 0.05; ANOVA; Figure 1E) whereas increased IL-6 release became evident only after 4 h (*P* < 0.001; ANOVA; Figure 1F). Treatment of primary glia with LPS (100 ng/ml) enhanced the expression of phosphorylated IκBα and c-jun between 10 and 30 minutes while phosphorylation of JAK2 and STAT1 was not apparent until 120 minutes (Figure 1G). No phosphorylation of JAK1 in response to LPS was apparent at any time point examined (Figure 1H, upper panel).

### Inhibition of JAK2 attenuates the LPS-induced phosphorylation of STAT1 and the release of pro-inflammatory cytokines TNFα and IL-6

**Figure 2:**
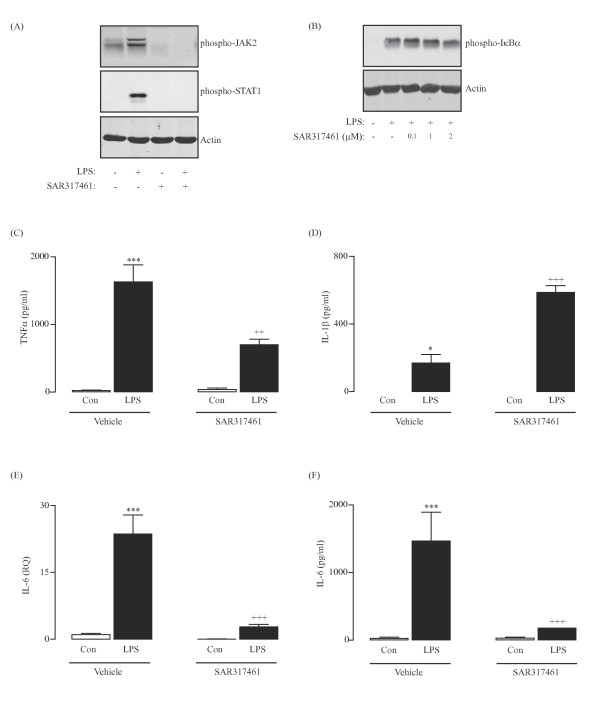
**Inhibition of JAK2 attenuates LPS-stimulated release of TNFα and IL-6 from glial cells.** A sample immunoblot indicates that expression of phosphorylated JAK2 and STAT1 were enhanced in glial cells treated with LPS (2 h; 100 ng/ml), and that this effect was attenuated when cells were incubated in the presence of a specific JAK2 inhibitor, SAR317461 (2 μM) (**A**). Expression of phosphorylated IкBα was enhanced in cells that were treated with LPS (**B**); however, co-incubation of cells with LPS and SAR317461 had no effect on the LPS-induced expression of phosphorylated IкBα (**B**). The LPS-stimulated release of TNFα at 6 h (**C**; ^***^P < 0.001; ANOVA; n = 3) was attenuated when cells were co-incubated with LPS and SAR317461 (^++^*P* < 0.01; ANOVA; n = 3), whereas inhibition of the LPS-induced expression of phospho-JAK2/STAT1 using SAR317461 enhanced the LPS-induced release of IL-1β at 9 h (**D**; ^+++^*P* < 0.001; ANOVA; n = 3). The LPS-induced up-regulation of IL-6 mRNA (**E**; ^***^P < 0.001; ANOVA; n = 3) was attenuated when cells were co-treated with SAR317461 (**E**; ^+++^*P* < 0.001; ANOVA; n = 3) as was the release of IL-6 (**F**; ^+++^*P* < 0.001; ANOVA; n = 3).

We used a specific JAK2 inhibitor, SAR317461, to evaluate the role of JAK2 in modulating LPS-induced changes. First, we confirmed that incubation of glia in the presence of LPS for 2 h increased JAK2 (upper panel; Figure 2A) and STAT1 (middle panel; Figure 2A) phosphorylation. Interaction of JAK proteins with their cognate receptors induces autophosphorylation of JAK. Predictably, treatment of glia with LPS and the specific JAK2 inhibitor, SAR317461, attenuated the LPS-induced enhancement of phosphorylated JAK2 and STAT1 (Figure 2A). No effect of SAR317461 was observed on the LPS-stimulated activation of NFкB signaling (Figure 2B). Additionally, SAR317461 had no effect on cell viability, even up to 5 μM concentration (data not shown). LPS significantly increased release of TNFα from glial cells at 6 h (*P* < 0.001; ANOVA; Figure 2C) and this was significantly attenuated when cells were incubated in the presence of SAR317461 (*P* < 0.01; ANOVA; Figure 2C). While the LPS-stimulated release of IL-1β at 6 h (*P* < 0.05; ANOVA; Figure 2D) was enhanced when cells were co-treated with SAR317461 (*P* < 0.001; ANOVA; Figure 2D), the LPS-induced increase in expression of IL-6 mRNA at 3 h (*P* < 0.001; ANOVA; Figure 2E) and release of IL-6 at 6 h (*P* < 0.001; ANOVA; Figure 2F) were significantly attenuated when JAK2 was inhibited (*P* < 0.001; ANOVA; Figure 2E,F).

### JAK2 associates with the TNFR1 and induces release of IL-6

**Figure 3:**
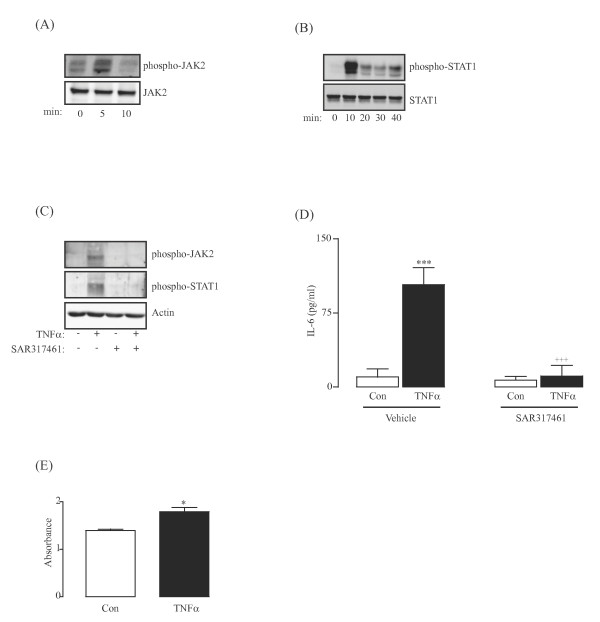
**JAK2 is phosphorylated and associates with TNFR1 in response to TNFα treatment.** TNFα receptor (TNFR1) was immunoprecipitated from cell lysates of glia that had been treated with TNFα (5 ng/ml) for 0, 5 or 10 minutes, and western-blotted using a phospho-JAK2 specific antibody. Incubation of glial cells with TNFα stimulated a rapid association between phosphorylated JAK2 and TNFR1 (**A**). TNFα treatment of glial cells also induced phosphorylation of STAT1 at 10 minutes (**B**). The TNFα-stimulated phosphorylation of JAK2 and STAT1 was attenuated when cells were co-incubated with SAR317461 (**C**). Representative immunoblots of three separate experiments are shown. Incubation of glial cells with TNFα (5 ng/ml) stimulated release of IL-6 at 6 h (**D**, ^***^P < 0.001; ANOVA; n = 3), an effect that was inhibited when cells were co-treated with TNFα and TG101209 (**D**, ^+++^*P* < 0.001; ANOVA; n = 3). TNFα had no adverse effect on cell viability after 24 h of treatment (**E**, *P < 0.05; Student’s *t*-test for independent means; n = 3).

JAK/STAT signaling is usually associated with cytokine receptors that lack intrinsic tyrosine kinase activity, and a number of cytokines mediate their signals through association with JAK2, including IL-6 and IL-12 [13,14]. The data from this study indicate that IL-6 was not released until 4 h after LPS treatment (Figure 1F), and IL-12 was not detected before 9 h following LPS treatment of glial cells (data not shown). In this study, the activation of JAK2 coincided with significant release of TNFα and, consequently, the effect of TNFα on JAK2 signaling was investigated. Glial cells were treated with TNFα and the interaction of TNFR1 and JAK2 was assessed by co-immunoprecipitation. TNFα treatment of glial cells enhanced the association between phosphorylated JAK2 and TNFR1 at 5 minutes (Figure 3A). Similarly, STAT1 phosphorylation was evident within 10 minutes of TNFα treatment (Figure 3B), and the TNFα-induced expression of phosphorylated JAK2 (Figure 3C, upper panel) and STAT1 (Figure 3C, middle panel) were inhibited when cells were co-incubated in the presence of SAR317461. Since TNFα was capable of inducing JAK/STAT signaling, we considered that the LPS-induced release of TNFα and subsequent activation of JAK2 may trigger release of IL-6. To assess this, glia were incubated with TNFα in the presence or absence of SAR317461 for 6 h. The data show that TNFα significantly increased IL-6 release (P < 0.001; ANOVA; Figure 3D) and that this effect was attenuated when cells were co-incubated with the JAK2 inhibitor (*P* < 0.001; ANOVA; Figure 3D). To ensure that the concentration of TNFα used here was not toxic, cells were incubated for up to 24 h with TNFα (5 ng/ml) and viability assessed using an (3-(4,5-dimethylthiazol-2-yl)-5-(3-carboxymethoxyphenyl)-2-(4-sulfophenyl)-2 H-tetrazolium (MTS) viability assay (Figure 3E). TNFα did not have any adverse effect on cell viability under the conditions used here (*P* < 0.05; Student’s t-test for independent means; Figure 3E).

### IL-6 modulates the LPS-stimulated release of IL-1β from glia

**Figure 4:**
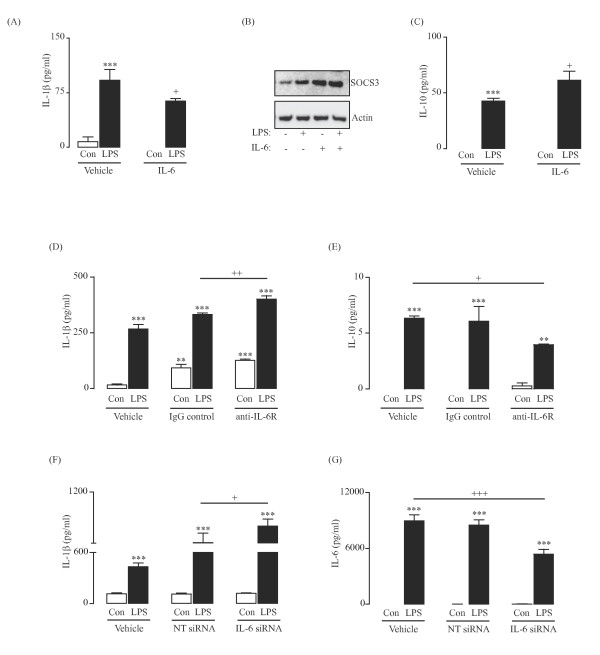
**IL-6 modulates the LPS-stimulated release of IL-1β from glia.** Glial cells were treated with LPS)(100 ng/ml) in the presence or absence of rat recombinant IL-6 (20 ng/ml) and cytokine concentrations were assessed at 24 h (**A,C**) and SOCS3 expression was examined at 90 minutes (**B**). The LPS-induced release of IL-1β (**A**; ^***^P < 0.001; ANOVA) was partially inhibited when cells were co-incubated with recombinant IL-6 (**A**; ^+^*P* < 0.05; ANOVA). SOCS3 expression was induced in the presence of LPS (**B**) and further enhanced when cells were co-treated with LPS and recombinant IL-6 (**B**). IL-10 production was stimulated by LPS treatment (**C**; ^***^P < 0.001; ANOVA), an effect that was greater when cells were stimulated with LPS in the presence of recombinant IL-6 (**C**; ^+^*P* < 0.05; ANOVA). Furthermore, inhibition of IL-6 signaling using a neutralizing antibody to the IL-6 receptor (IL-6R) exacerbated the LPS-induced effect on IL-1β release (**D**; ^++^*P* < 0.01; ANOVA) compared to the IgG istoype control antibody while inhibiting the LPS-stimulated release of IL-10 (**E**; ^+^*P* < 0.05; ANOVA). Similarly, when the LPS-stimulated production of IL-6 was inhibited using siRNA targeted against IL-6 (**G**; ^+++^*P* < 0.001; ANOVA), the LPS-induced release of IL-1β was enhanced (**F**; ^+^*P* < 0.05; ANOVA), effects that were not apparent in cells that were incubated in the presence of LPS and the non-target (NT) siRNA (**F,G**).

It has been reported that IL-6 can act as a switch between innate and adaptive immunity [15] and that it can also act as an anti-inflammatory cytokine under certain conditions [16-19], perhaps by modulating the balance between pro- and anti-inflammatory cytokines. We considered that IL-6 might impact on the LPS-induced release of IL-1β at later time-points and, to investigate this, cells were incubated for 24 h with LPS in the presence or absence of recombinant IL-6. LPS significantly increased the release of IL-1β (*P* < 0.001; ANOVA; Figure 4A) and recombinant IL-6 partially attenuated its release ( P < 0.05; ANOVA; Figure 4A). One mechanism by which IL-6 might modulate IL-1β production is by increasing expression of suppressor of cytokine signaling (SOCS) proteins. Here we show that the LPS-associated increase in SOCS3 expression, which was evident at 90 minutes, was enhanced by IL-6 (Figure 4B, upper panel).

A second possible mechanism by which IL-6 may modulate IL-1β release is by increasing anti-inflammatory cytokines such as IL-10. We demonstrate that co-treatment of glial cells with recombinant IL-6 enhanced the LPS-induced release of IL-10 at 24 h (*P* < 0.05; ANOVA; Figure 4C).

To further investigate the effect of IL-6 on glial cell production of IL-1β, IL-6 signaling was down-regulated by incubating cells with LPS in the presence or absence of a neutralizing antibody to the IL-6 receptor or an isotype (IgG) control (Figure 4D,E). The LPS-stimulated production of IL-1β (*P* < 0.001; ANOVA; Figure 4D) was enhanced when cells were co-treated with a neutralizing antibody to the IL-6 receptor compared to treatment with the IgG control antibody (*P* < 0.01; ANOVA; Figure 4D), whereas the LPS-induced effect on IL-10 production ( P < 0.05; ANOVA; Figure 4E) was attenuated ( P < 0.05; ANOVA; Figure 4F). Consistently, inhibition of the LPS-induced release of IL-6 using siRNA targeted to *IL6* (Figure 4F,G) enhanced the LPS-induced release of IL-1β (*P* < 0.05; ANOVA; Figure 4F).

## Discussion

The purpose of the present study was to examine the factors which impact on time-related release of inflammatory cytokines following LPS stimulation focusing, in particular, on the role of the JAK2/STAT1 signaling pathway. The data show that LPS-induced TNFα triggers activation of JAK2/STAT1, and reveal a role for JAK2 activation in the LPS-stimulated release of IL-6 which, in turn, modulates IL-1β production.

SAR317461, a small molecule inhibitor of JAK2 identified by structure-based drug design [20], was used as it has been shown to potently down-regulate expression of phosphorylated JAK2 and STAT1 without affecting expression of total JAK2 and STAT1 in multiple myeloma cell lines [21]. Here we show that SAR317461 potently inhibits phosphorylation of JAK2 itself along with the JAK2 substrate STAT1 in LPS-stimulated glial cells. A similar effect has been reported in hematopoietic progenitor cells [22] and lung cancer cells [23]. LPS did not affect phosphorylation of JAK1 in glia, indicating a specific role for JAK2 in LPS-stimulated changes.

A primary role for JAK2 was identified in the LPS-stimulated release of IL-6, since co-treatment of glia with LPS and SAR317461 attenuated IL-6 release. In agreement with previous reports [15], the LPS-induced release of TNFα from glia preceded release of IL-6, which became detectable in supernatant after 4 hours. IL-6 lacks a transmembrane domain, and is transported to the plasma membrane to enable constitutive release, consequently intracellular IL-6 concentration is very low [24]. This suggests that LPS-stimulated release of IL-6 occurs as a result of enhanced transcription of IL-6, and this is consistent with the observation that IL-6 mRNA was increased 2 hours after LPS stimulation preceding detection of IL-6 in the supernatant. The requirement for transcription in driving the LPS-induced IL-6 release is supported by the demonstration that SAR317461 also inhibited the LPS-induced up-regulation of IL-6 mRNA.

Other signaling molecules, including NFкB, are activated downstream of TNFα receptor activation [25-27]. NFкB activation is also known to affect IL-6 production/release in response to LPS treatment. Inhibition of JAK2/STAT1 exerted no effect on the NFκB signaling complex, although it inhibited LPS-induced IL-6 production. Crosstalk between NFκB and JAK/STAT signaling pathways has been reported [28-30], and it is therefore possible that NFкB-associated modulation of IL-6 release reported in the literature may occur as a result of this crosstalk rather than as a direct effect of inhibition of NFκB-dependent gene transcription.

Both transcription of TNFα and its release were up-regulated in LPS-stimulated glial cells. Inhibition of JAK2 modestly attenuated the LPS-induced release of TNFα, contrasting with its ability to completely block the LPS-induced release of IL-6. However, the mechanism underlying TNFα release differs from that of IL-6. Translocation of TNFα to the membrane is required. Here it must undergo a cleavage event prior to release [31] and, consequently, TNFα release may be modulated by regulating processing enzymes, up-regulating transcription or release of upstream pro/anti-inflammatory mediators.

Activation of JAK2/STAT1 is generally observed within 15 minutes of receptor stimulation [32]. A somewhat delayed LPS-induced response was observed here, indicating that activation of JAK2 occurs downstream of signaling kinases or secondary to LPS-induced release of other cytokines. IL-6 and IL-12 are examples of cytokines which activate JAK2 [13,14], but JAK2 activation preceded LPS-induced release of both of these cytokines from glia. In contrast, TNFα release roughly coincided with the activation of JAK/STAT signaling. While TNFR1 does not contain any intrinsic tyrosine kinase activity, TNFα is known to stimulate tyrosine phosphorylation [33-37] and here we show that treatment of glia with TNFα induced an association of phosphorylated JAK2 with TNFR1. A similar association between JAK2 and the receptor has been reported in HEK293, H1299 lung adenocarcinoma and MCF7 human breast carcinoma cells [38]. However, to our knowledge, no such observation has been made in cells from the CNS. In the present study, the TNFα-induced association of activated JAK2 and TNFR1 led to STAT1 phosphorylation and release of IL-6; SAR317461 prevented both of these changes. SAR317461 had no effect on phosphorylated IкBα, ruling out any role for NFкB. Interestingly a TNFR1 deficiency has a protective effect on myocardial tissue and decreases myocardial IL-6 concentrations [39], an observation attributed to enhanced SOCS3 and decreased STAT3 activation.

**Figure 5:**
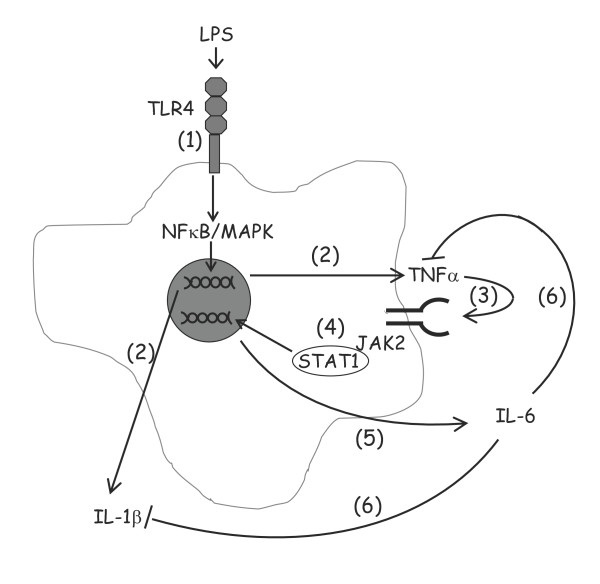
**Schematic representation of LPS-induced signaling and cytokine release.** Toll-like receptor (TLR)4 is stimulated by LPS and triggers activation of MAPKs and NFkB (1) which results in release of TNFα and IL-1β (2). Released TNFα interacts with its receptor (3), activating JAK2 and STAT1 (4) which up-regulates transcription and release of IL-6 (5). IL-6 feeds back to inhibit further release of TNFα and IL-1β (6), limiting the inflammatory changes.

LPS-induced release of IL-6 from glia occurs later than that of TNFα and IL-1β. IL-6 exerts pro- and anti-inflammatory effects within the CNS [16-19,40-44]. In the absence of SOCS3, IL-6 inhibits the LPS-stimulated up-regulation of IL-1β mRNA as well as the release of TNFα and IL-12 p40 from macrophages [45]. Interestingly, LPS-induced up-regulation of SOCS3 in macrophages was enhanced by IL-6. This result indicates an inhibitory effect of LPS/IL-6 co-treatment on JAK/STAT signaling, and may explain the partial attenuation of IL-1β release observed when cells were co-incubated with LPS and recombinant IL-6. Conversely, the LPS-induced release of IL-1β was enhanced when IL-6 release was inhibited with siRNA targeted against the IL-6 gene or when the interaction between IL-6 and its receptor was disrupted by an IL-6R monoclonal antibody. However, we show that incubation of cells in the presence of IL-6 for 24 h also increases the anti-inflammatory cytokine, IL-10; this, like SOCS3, plays a role in modulating IL-1β release from several cells including microglia [46]. The findings indicate that LPS induces production and release of TNFα which leads to activation of TNFR1 and the association between this receptor and JAK2. The subsequent phosphorylation of JAK2 triggers STAT1 activation and increased production and release of IL-6 (Figure 5). We propose that two of the events that occur as a result of increased IL-6 are up-regulation of SOCS3 and production of IL-10, both of which contribute to the longer term modulation of IL-1β production. Whether the proposed cascade of events is occurring in one specific cell type is unclear. LPS-induced activation of JAK/STAT and the release of IL-6 was apparent in both isolated astrocytes and microglia, an effect that could be modulated by SAR317461 (data not shown). The subsequent effects of modulating IL-6 signaling were not investigated in isolated cell types since it is uncertain whether astrocytes express the IL-6 receptor or require a soluble form to be released to confer IL-6 responsiveness [10].

Neuroinflammation has been identified as a key process in driving the pathogenesis associated with a number of neurodegenerative conditions such as Alzheimer’s disease, multiple sclerosis, Parkinson’s disease and brain injury. Microglia and astrocytes are pivotal in driving this process since they release inflammatory mediators. Therefore, strategies which target specific components of the pathways leading to release are likely to be important in uncovering the sequence of events underlying the pathogenesis of these diseases. Data from this study identify a key regulatory role for JAK2/STAT1 signaling in the production and release of IL-6 from glial cells, and highlight the complex interactions which govern time-related responses to LPS.

## Abbreviations

ANOVA, analysis of variance; DMEM, Dulbecco’s Modified Eagle’s Medium; ELISA, enzyme-linked immunosorbent assay; IL, interleukin; JAK, Janus kinase; LPS, lipopolysaccharide; MAPK, mitogen-activated protein kinases; NFκB, nuclear factor κB; NT, non-target; SOCS, suppressor of cytokine signaling; STAT, signal transducers and activators of transcription; TNF, tumor necrosis factor; TNFR1, TNFα receptor.

## Competing interests

The authors declare that they have no competing interests.

## Authors’ contributions

AM designed and planned the study and carried out the cell culture, cell treatments, cytokine release and mRNA analysis, protein analysis and immunoprecipitation experiments and drafted the manuscript. JB participated in the cytokine release and mRNA expression temporal profile analysis. ML designed and planned the study and helped to draft the manuscript. All authors read and approved the final manuscript.
